# Playful Interventions Increase Knowledge about Healthy Habits and Cardiovascular Risk Factors in Children: The CARDIOKIDS Randomized Study

**DOI:** 10.5935/abc.20170107

**Published:** 2017-09

**Authors:** Fátima H. Cecchetto, Daniela B. Pena, Lucia C. Pellanda

**Affiliations:** 1 Pós-Graduação em Ciências da Saúde: Cardiologia - Instituto de Cardiologia/Fundação Universitária de Cardiologia, Porto Alegre, Brazil; 2 Universidade Federal de Ciências da Saúde de Porto Alegre, Porto Alegre, Brazil

**Keywords:** Child, Pediatric Obesity, Motor Activity, Games, Recreational, Knowledge, Randomized Controlled Trial as Topic

## Abstract

**Background:**

Childhood obesity is an important health problem worldwide. In this context, there is a need for the development and evaluation of innovative educational interventions targeting prevention and formation of health habits.

**Objectives:**

To ascertain the impact of ludic workshops on children’s knowledge, self-care, and body weight.

**Methods:**

This was a randomized, clinical study with 79 students aged 7-11 years, conducted from March to November 2012. Anthropometric measurements were collected and two questionnaires (Typical Day of Physical Activities and Food Intake, in Portuguese, and the CARDIOKIDS, a questionnaire of knowledge about cardiovascular risk factors) were applied at baseline, at the end of intervention, and three months thereafter. The intervention consisted of eight playful workshops, which involved the presentation of a play.

**Results:**

Seventy-nine students were randomized to the intervention (n = 40) or the control group (n = 39). Mean age was 10.0 ± 1.1 years. After eight weeks, the intervention group showed significant improvement in the knowledge score (p < 0.001). There was an increase in physical activity scores in both groups, but with no difference between the groups at the end of intervention (p = 0.209). A reduction in the BMI percentile was observed in the intervention group, but there was no significant statistical difference between the two groups after the intervention.

**Conclusions:**

Playful interventions may improve knowledge and physical activity levels in children and, when combined with other strategies, may be beneficial to prevent child obesity and improve self-care.

## Introduction

Childhood obesity is an important health problem worldwide.^1,2^ A study including 144 countries projected an increase of excess weight from 4.2% in 2010 to 9.1% in 2020, representing 60 million children; of those, 35 million will be from developing countries.^3,4^

Although genetic factors can influence the susceptibility to weight gain, the consensus is that a sedentary lifestyle, inadequate dietary practices and changes in family structure all contribute to this epidemic.^2^ Urbanization and other environmental factors bring profound habits changes, especially regarding eating habits and physical activity.^5,6^ In Brazil, economic and media globalization contributed to significant changes regarding diet (with more spread use of processed and ultraprocessed foods in detriment to more traditional preparations) and family habits, such as having all the meals together.^3^

The number of children between 5 and 9 years with excess weight more than doubled in the country from 1989 to 2009^3^, escalating from 15% to 34.8%, while the number of obese children of the same age increased 30%, from 4.1% to 16.6%.^4^

Studies have shown an association between childhood obesity and risk factors for the development of chronic illnesses such as diabetes mellitus, hypertension, dyslipidemia and other cardiovascular diseases.^7-11^ Therefore, there is an urgent need to focus on early prevention. Early health promotion strategies with comprehensive nutritional and physical activity guidance have shown to increase knowledge and improve self-care among patients with chronic conditions.^1,12,13^ However, studies regarding educational interventions for obesity prevention in children are heterogeneous and yield different results.^14-20^ Recent meta-analysis of educational interventions for obese and non-obese children showed positive results regarding blood pressure and waist circumference reduction, but a less clear effect on body mass index (BMI).^18,19^

In this context, there is a need for development and evaluation of innovative educational interventions targeting prevention and health habit formation. We designed a low-cost educational intervention based on playful workshops for children, in a low resource setting, that could be useful in many contexts worldwide. The objective of the present study is to ascertain the impact of this intervention on children's knowledge, physical activity levels and BMI in a low resource community in a developing country.

## Methods

This was a cluster randomized, controlled study, carried out from March to November 2012. Seventy-nine students in four classes participated in the study and were randomly divided into two groups with two classes each, 40 participants in the intervention group and 39 students in the control group. The study was registered in the Brazilian Clinical Trials Registry under the code RBR-8f6wr7 (http://www.ensaiosclinicos.gov.br/rg/RBR-8f6wr7/). All parents signed the informed consent.

### Participants

Inclusion criteria were children from seven to eleven years of age, who attended a philanthropic program during non-school hours for children with low socio-economic conditions in the city of Porto Alegre in the southern Brazilian state of Rio Grande do Sul. All children were healthy and were also enrolled in regular schools.

Exclusion criteria were clinical diseases that would prevent participation in the program or anthropometric evaluation. No children had any of these conditions, and therefore there were no exclusions after the signing of the consent form.

After randomization, 6 children in the intervention group and 5 in the control group were lost to follow up, because they moved to other schools or refused to continue in the study, thus resulting in 40 children in the intervention group and 39 in the control group. All other children attended to all sessions and completed the study. For those children that could not attend in a specific day, another day was scheduled.

### Randomization

A table with random numbers representing each class was created by an investigator who was not related to the study, with the aid of the randomization tool available at www.randomization.com. These numbers were placed and sealed in brown envelopes. After inclusion of all participants, an investigator who was not related to the study opened the envelopes and the classes were assigned to the arms of intervention or control (cluster randomization).

### Interventions

Intervention consisted of eight weekly Playful workshops lasting between 30 and 60 minutes during 60 to 90 minutes each. The workshops included collage, painting, games creation, physical activity, music and dance, and simulations of real life situations, all involving the importance of healthy habits for heart health, especially relating to healthy foods and physical activity. The same investigator (FHC, a Registered Nurse) performed all the activities in the classroom or in the school patio. The workshops are described in box 1.

**Box 1:** The workshops.

Workshop 1	Students were divided in small groups of four. The task proposed to the groups was to make collages depicting healthy and unhealthy foods using old magazines, always with the guidance of the tutor. After completing the tasks, all groups discussed on the subject.
Workshop 2	Children played the role of a healthy heart and "all the things a happy heart enjoys" in a play developed by the group.
Workshop 3	The students discussed the importance of physical activity and with drawings and other materials, represented the activities that they liked most.
Workshop 4	Drawing and collages about healthy and unhealthy foods
Workshop 5	Dance class with music.
Workshop 6	Students showed some of the physical activities they liked and discussed in groups ways to perform them more frequently.
Workshop 7	Children built a "memory game" from recycled materials. The game contained pictures of healthy foods and different physical activities. This material was later used every day by the teacher in classes, for about 10 minutes.
Workshop 8	The investigator brought to class foods such as fruits, chocolate, vegetables, oil, eggs, salt and sugar. For each food, the group discussed its properties and if it was healthy or unhealthy. The students drew little happy or unhappy hearts accordingly. In all cases, it was discussed that all foods have good and "not so good" characteristics, and it is important to be aware of the quantities and frequency of consumption.

Source: Cecchetto,Pellanda 2013

The control group maintained their usual activities in math, language and music with their teacher in their class in the same period. They also had their usual physical education classes, including soccer, capoeira and tennis.

### Outcomes

The primary outcome considered was increased knowledge about healthy habits and risk factors for cardiovascular disease, measured by the CARDIOKIDS questionnaire (Portuguese validated version, see below) immediately after and four weeks after intervention.

Secondary outcomes were change in physical activity levels and body mass index immediately after intervention.

### Instruments

Two structured questionnaires were used in this study.

The Portuguese version of the "typical day of physical activity and food intake" (DAFA, *Dia Típico de Atividades Físicas e de Alimentação*) is an illustrated and structured questionnaire developed by a group of Brazilian researchers with the objective to obtain information about weekly habits of physical activity in children aged seven to eleven years^21^. The instrument contains 36 illustrations of physical activities in different intensities, and a score system was developed to summarize the answers. Of a total of 141 points, values below 36 are classified as "less active," 37 to 58 as "intermediate," and 59 to 141 as "more active".

The questionnaire regarding knowledge of healthy habits and risk factors for cardiovascular disease (CARDIOKIDS) was also developed in Brazil and validated for children from 7 to 11 years. It contains twelve illustrated questions, divided in two dimensions: healthy habits (healthy eating and physical activity) and risk factors for cardiovascular diseases. Response options consist of three faces: "happy" (good for the heart), "unhappy" (bad for the heart), and "neutral" (do not know). Scores of 11-12 correct answers were considered as "excellent knowledge," 8-10 correct answers were considered "good knowledge," and scores below 7 correct answers were considered "insufficient knowledge".^22^

### Data collection

Data collection took place at three moments between March and November of 2012, beginning soon after obtaining the informed consent from their parents.

At baseline, anthropometric measurements (weight and height) were performed and two questionnaires (DAFA and CARDIOKIDS) were applied. The same parameters were measured just after the intervention. Twelve weeks after completion of the program, the CARDIOKIDS questionnaire was repeated to evaluate knowledge retention.

Anthropometric measurements were obtained according to the recommendations of the World Health Organization.^23^ For weight measurement, the participants were asked to remove their shoes and heavy clothing. A Plenna Wind digital scale was used, with a maximum capacity of 150 kg, accuracy of 100 g, and a stadiometer with a measuring range of 192 cm. Weight and height were measured twice by one of the investigators. Children above the 85th BMI percentile were considered overweight, and above the 95th were considered obese.^24^

### Statistical analysis

The sample size was based on previous results from a pilot study on 38 individuals, in which an average of eight correct answers in the CARDIOKIDS questionnaire was observed, with standard deviation of 2.0. We estimated a 30% increase in the knowledge scores in the intervention group with a power of 95% and 0.05 level of significance, yielding a minimum sample of 44 participants (22 in each group). Considering possible losses during the study and the cluster effect, a total sample of 40 participants for each group was planned. Data analysis and processing was performed using the IBM SPSS Statistics software, version 14.0. Continuous variables are expressed by means and standard deviations and categorical variables are expressed by absolute and relative frequencies. Normality of data was evaluated with histograms and the Kolmogorov-Smirnof test. For comparisons between groups after the intervention we used the paired Student t test for continuous variables and the chi-squared test for categorical variables. GEE (generalized estimating equations) were used for comparisons between groups and within groups across different periods (baseline, immediately after and 12 weeks after intervention) adjusting for age and gender. The Bonferroni adjustment was used to identify differences in paired analysis. Repeated measures ANOVA was used to compare different points in time between groups. In all comparisons, a p < 0.05 was considered significant.

## Results

The institution receives a total of 185 students from 7 to 11 years old. Parents or guardians of 25 children did not sign the consent form, and 70 children did not meet the inclusion criteria, thus resulting in a total sample of 90 children, 46 randomized in the intervention group and 44 in the control group.

**Figure 1:**
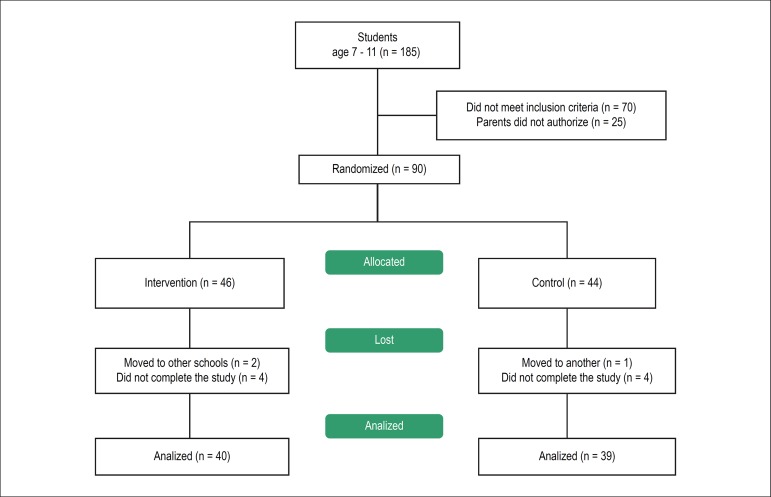
CONSORT ﬂow chart depicting recruitment and randomization of the children into the study groups.

Figure 1 presents the flow chart according to consort recommendations (www.consort-statement.org.). Table 1contains baseline characteristics of the intervention and control groups. Students in the control group were older (p = 0.007) and more likely to be in grades 5-6 (p < 0.001) than students in the intervention group. Most children were classified as less active.

**Table 1:** Baseline characteristics of students in the intervention and control groups

Participants (n = 79)	Intervention (n = 40)	Control (n = 39)	p
Age mean ± SD	*9.3 ± (1.4)	10.03 ± (1.1)	0.007
**Gender n (%)**			0.730
Female	*20 (50%)	22 (56.4%)	
Male	20 (50%)	17 (43.6%)	
**Ethnicity n (%)**			**1.000
Caucasian	30(75%)	29(74.4%)	
African	10(25%)	10(25.6%)	
**Current school grade n (%)**			0.001
Grades 2-4	23 (57.5%)	10 (25.7%)	
Grades 5-6	17 (42.5%)	29 (74.4%)	
Height (cm) mean ± SD	136.9±(11.5)	141.7 ± (8.7)	***0.041
Weight (kg) mean ± SD	36.7 ± (12.5)	38.1 ± (10.6)	0.571
BMI (km/m^2^) mean ± SD	19.1±(3.8)	19.2 ± (4.5)	0.875
**Nutritional status n (%)**			0.952
< 85^th^ percentile	25(62.5%)	24(61.5%)	
≥ 85^th^ percentile (overweight)	6(15%)	7(18%)	
> 95^th^ percentile (obese)	9(22.5%)	8(20.5%)	
**Physical activity (DAFA classification) n (%)**			0.209
Less active	30(75%)	28(71.8%)	
Intermediate	8(20%)	10 (25.6%)	
More Active	1(5%)	1(2.7%)	

Continuous variables are expressed by means and standard deviations and categorical variables are expressed by absolute and relative frequencies.

** chi-squared test.

*** paired t test.

Source: Cecchetto, Pellanda 2014^22^

**Figure 2:**
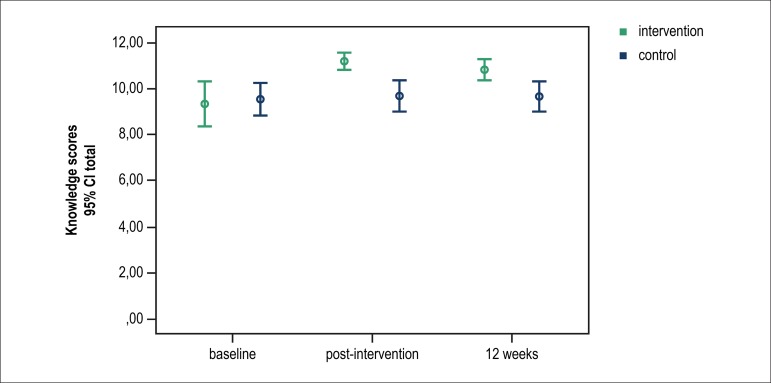
Intra- and between-group comparisons of knowledge at baseline, immediately after and at 12 weeks after the intervention. P-value for differences between the groups: 0.016; P-value for differences immediately after and 12 weeks after the intervention as compared with baseline in the intervention group: < 0.001; P for differences immediately after and 12 weeks after the intervention as compared with baseline in the control group group: 0.337; P-value for interaction between group and time: 0.002

Table 2 presents the results for knowledge scores before and after the educational intervention. The results demonstrate that both groups had good knowledge before the intervention period, according to both dimensions of the questionnaire. However, there was a significant difference between the groups after intervention due to an increase in the scores of the intervention group. In the 12^th^ week of evaluation, the results showed a reduction in the intervention group's knowledge scores, but still a significant difference in relation to the control group (Figure 2).

**Table 2:** Knowledge dimension before, immediately after and after 12 weeks of intervention or control activities

		Before intervention	After intervention	12 weeks after intervention	p *	Variation before-immediately after (95%CI)	Variation before-12 weeks after (95% CI)
		Mean(SE)**	Mean(SE)**	Mean(SE)**			
Dimension:Health habits	Intervention(n = 40)	4.2(0.3)	5.6(0.2)	5.2(0.2)	< 0.001	1.4(0.9 - 2.0)	1.0(0.3 - 1.6)
	Control(n = 39)	4.1(0.3)	4.1(0.2)	4.1(0.2)		0.02(-0.6 - 0.6)	0.04(-0.6 - 0.7)
Dimension:Risk factors	Intervention(n = 40)	5.2(0.2)	5.6(0.1)	5.7(0.1)	0.129	0.5(-0.01 - 1.0)	0.5(0.03 - 1.0)
	Control(n = 39)	5.3(0.2)	5.4(0.1)	5.4(0.1)		0.04(-0.5 - 0.5)	0.04(-0.5 - 0.5)

* Interaction between group and time - Anova -repeated measures.

** Adjusted means (age and gender) and standard errors (SE).

Source: Cecchetto,Pellanda 2014^22^

Table 3 shows the results for BMI percentiles and physical activity pre- and post-intervention. There were no differences for BMI percentiles. After the intervention, both groups showed a significant increase in physical activity level from baseline (p < 0.001), but there was no significant difference between groups after intervention (p = 0.804).

**Table 3:** Comparison between groups before and after the intervention period: BMI and Physical activity score, n = 79

Variables	Intervention (n = 40) Mean (95% CI)	Control (n = 39) Mean (95% CI)
**BMI (kg/m^2^)**		
Before	70.9 (62.5; 79.3)	62(52.0; 72.0)
After	69.9 (61.9; 77.9)	63.8 (54.5; 73.1)
Mean difference (CI 95%)	- 1.0 (-3.7; 1.6)	1.8 (-1.0; 4.8)
Difference between groups: p = 0.240** Interaction between group and time: p = 0.669		
**Physical Activity (Score)**		
Before	31.9 (26.7; 37.1)	27.3 (22.6; 32.0)
After	34.1 (29.2; 39.0)	29.7 (24.9; 34.5)
Mean difference (95 CI%)	2.2 (1.0; 3.4)	2.4 (1.5; 3.3)
Difference between groups: p = 0.201 Interaction between group and time: p = 0.804		

** chi-squared test. BMI: body mass index.

Source: Cecchetto,Pellanda 2014^2^

## Discussion

This randomized controlled trial showed that an educational intervention based on playful activities was effective in increasing physical activity scores and knowledge about healthy habits and risk factors for cardiovascular disease.

The school environment is considered to be a good setting for health promotion because it allows reaching children and adolescents.^11^ Playful activities focusing on health themes are an opportunity to create a bond to facilitate the sharing of experiences and knowledge, empowering a child to take care of the own health.

This is increasingly important in a context of rising prevalence of chronic diseases and unhealthy habits very early in life. In accordance with this context, approximately 38% of this sample of school children was overweight.^7,25^ In south Brazil, 28% of children aged 11-18 years were overweight and 10% were obese.^6^

In our study, both groups had previous knowledge of healthy habits and cardiovascular risk factors before the educational interventions, with no significant difference between groups at baseline. One possible explanation for these results is the fact that schools and the media have recently become more concerned about providing guidance on nutrition and the importance of physical activity for improved quality of life and prevention of cardiovascular diseases and obesity. However, these data suggest that playful activities may be a good educational strategy for students, and that knowledge about healthy habits and risk factors for cardiovascular diseases is present among students in this age group.

One recent study carried out with teachers and students aged 5 to 10 years in the Brazilian capital city of Brasília showed that, after nutritional interventions, there was an increase of knowledge from 61% to 74% in children, and a similar increase among the teachers.^26^ One study carried on students with higher BMIs out in the southern region of Brazil in 2005 showed that these students have less knowledge and less healthy dietary practices than those observed in the present study.^27^ Another recently-published study performed with 464 students in northern Portugal from November 2008 to March 2009, using interventions based on the Model of Health Promotion and cognitive theory, showed satisfactory results in relation to changes in dietary habits of the children in the intervention group.^28^ Thus, it is believed that an increased knowledge can improve self-care related to weight control and dietary habit changes.

The evaluation of BMI percentiles did not show a statistically significant difference between the groups at the end of the intervention period, but a slight reduction in percentile was observed in the intervention group, along with a small increase in the control group. Studies involving interventions in schools for prevention and treatment of obesity have shown controversial results,^19^ especially in non-selected populations composed of normal weight and overweight children. In these conditions, it is more difficult to observe changes in BMI, since a large part of the population does not need to lose weight. The heterogeneity of the interventions regarding type, duration and number of activities included must also be considered.^19,29^ It is also possible that more prolonged and comprehensive interventions show more positive results regarding BMI changes.^30^ Analysis of baseline data showed that the two groups were homogenous in regards to sex, ethnicity, weight, physical activity and knowledge, but the intervention group was older than the control group.^31^

It is also important to emphasize that BMI is a controversial measure to be pursued as an outcome in children. It is insensitive to changes in body composition (for example, gain of lean mass) and does not account for possible ethnic or body type differences. Additionally, the most important outcomes in children may be permanent habit change, and not BMI.

In regard to physical activity, our results are similar to various other studies, in which the majority of this population is considered to have low levels of physical activity. Others studies carried out in 2004 in others regions Brazil, showed that 40% to 67% of children and 61% of the adolescents were sedentary.^29,32^ Lifestyle changes of families and students, in which television, videogames and the computer have become the greatest source of entertainment among children and youth contribute to these numbers. Additionally, urban violence, especially in low-income settings, has been described as a barrier for children to become involved in sports and other outdoor activities.^32^

After intervention, we observed an increase in physical activity in both groups. This might be due to contamination (children in the intervention group who became more active may have influenced children in the control group) or to climate changes. While the intervention started during the winter, which is rainy and with temperatures around 10ºC in Porto Alegre, and the second evaluation was performed during warmer weather.

Programs implemented with students to increase their physical activity level and provide dietary guidance have shown good results,^1,32^ but controversies exist regarding the best intervention to be applied. This may be due to the fact that these programs are applied to various groups with many cultural and environmental differences and interventions need to be customized according to these factors.

It must also be emphasized that knowledge is fundamental to motivate change, but is not enough to provoke persistent change. Education and health strategies involving playful activities can improve self-care, but must be done in conjunction with other strategies. Improving knowledge is the first step in any comprehensive prevention strategy, empowering the child for taking care of the own health.

### Limitations of the study

Some limitations of the study deserve mention. First of all, the study was carried out in an institution with low socioeconomic resources, making it difficult to apply these data to other student populations. The second limitation is related to the non-participation of students' parents in the study, as recent studies report success with strategies that include family members. Also, the intervention period of eight weeks may be too short to observe a significant change in habits resulting in weight loss. Finally, there was a difference of schooling between groups, with more children in the control group belonging to more advanced grades. However, this difference would reduce the differences between groups after intervention, thus altering the results to an opposite direction than that of our hypothesis.

## Conclusion

Our results show that a simple, low-cost intervention consisting of playful educational activities performed with low-income children in a school may help improving knowledge about healthy habits and risk factors for cardiovascular disease, and may be useful for the planning of preventive strategies in similar settings.
